# Pathobiology of *Avian avulavirus 1*: special focus on waterfowl

**DOI:** 10.1186/s13567-018-0587-x

**Published:** 2018-09-19

**Authors:** Zaib Ur. Rehman, Chunchun Meng, Yingjie Sun, Khalid M. Mahrose, Sajid Umar, Chan Ding, Muhammad Munir

**Affiliations:** 1 Shanghai Veterinary Research Institute (SHVRI), Chinese Academy of Agricultural Sciences (CAAS), Shanghai, 200241 China; 2 Department of Poultry Science, Faculty of Veterinary and Animal Sciences, PMAS Arid Agriculture University, Rawalpindi, 46300 Pakistan; 3 Jiangsu Co-innovation Center for Prevention and Control of Important Animal Infectious Diseases and Zoonoses, Yangzhou, 225009 China; 4 Shanghai Key Laboratory of Veterinary Biotechnology, Shanghai, 200241 China; 5 Poultry Department, Faculty of Agriculture, Zagazig University, Zagazig, 44511 Egypt; 6 Biomedical and Life Sciences, Lancaster University, Lancaster, LA1 4YG UK

## Abstract

*Avian avulaviruses serotype* 1 (abbreviated as APMV-1 for the historical name avian paramyxovirus 1) are capable of infecting a wide spectrum of avian species with variable clinical symptoms and outcomes. Ease of transmission has allowed the virus to spread worldwide with varying degrees of virulence depending upon the virus strain and host species. The emergence of new virulent genotypes from global epizootics, and the year-to-year genomic changes in low and high virulence APMV-1 imply that distinct genotypes of APMV-1 are simultaneously evolving at different geographic locations across the globe. This vast genomic diversity may be favoured by large variety of avian species susceptibility to APMV-1 infection, and by the availability of highly mobile wild birds. It has long been considered that waterfowls are not sensitive to APMV-1 and are unable to show any clinical signs, however, outbreaks from the 90′s contradict these concepts. The APMV-1 isolates are increasingly reported from the waterfowl. Waterfowl have strong innate immune responses, which minimize the impact of virus infection, however, are unable to prevent the viral shedding. Numerous APMV-1 are carried by domestic waterfowl intermingling with terrestrial poultry. Therefore, commercial ducks and geese should be vaccinated against APMV-1 to minimize the virus shedding and for the prevention the transmission. Genetic diversity within APMV-1 demonstrates the need for continual monitoring of viral evolution and periodic updates of vaccine seed-strains to achieve efficient control and eradication of APMV-1 in waterfowls.

**Electronic supplementary material:**

The online version of this article (10.1186/s13567-018-0587-x) contains supplementary material, which is available to authorized users.

## Introduction

Newcastle disease (ND) is one of the most devastating and commonly prevalent diseases in the poultry industry, around the world. Owing to immense economic losses, World Organization for Animal Health has categorized the disease as “notifiable” [1]. The disease outbreaks are enormous and the host spectrum is broad, thus making ND as one of the primary limiting factor in the development of the poultry industry, especially in the developing countries [2, 3]. It is caused by the *Avian avulavirus 1* (APMV-1), which belongs to the *Avulavirus* genus within *Paramyxoviridae* family. All APMV-1 strains can be classified into velogenic (highly virulent), mesogenic (intermediate virulent) and lentogenic (non-virulent) based on the intracerebral pathogenicity index (ICPI) in day old specific pathogen free (SPF) chickens [4, 5] or nature of three amino acids at the position 113–116 in the un-cleaved fusion protein cleavage site. APMV-1 is considered to be virulent, if these amino acids are basic in nature with a phenylalanine at position 117, and having ICPI value of 0.7 in 1 day old chicks [3, 6].

APMV-1 is a single-stranded, negative-sense, non-segmented and enveloped RNA virus with genome length of 15.2 kb. APMV-1 genome encodes for six co-linear genes that translate into six proteins and two non-structural proteins. Structural proteins include nucleoprotein (NP), matrix (M), fusion (F), hemagglutinin–neuraminidase (HN), phosphoprotein (P) and large RNA-dependent RNA-polymerase (L). The RNA editing of the P gene can result in the expression of V and W only in the virus infected cells [7–10]. Virulence of APMV-1 varies and depends upon the host species, and chicken and turkey are more susceptible than ducks and geese.

Generally, waterfowls were considered to be the natural reservoir for APMV-1 [1]; commonly for lentogenic APMV-1 [11]. Traditional view on the resistance of waterfowl against APMV-1 has been challenged since the report of continuous outbreaks in different provinces of china in goose (1997) [12, 13] and ducks (2002) [14]. Number of clinical ND outbreaks are increasing in the waterfowls [15–18]. Outbreaks of ND in ducks and geese indicate that these are not only the carrier, but also show clinical outcome of the disease. Factors that led to the change in the pathogenic spectrum of APMV-1 in waterfowl remain elusive. Investigation of the molecular mechanisms of increased pathogenicity of APMV-1 in waterfowls would provide foundations in designing any control strategies for the disease, as well as to improve health and welfare standards of the waterfowl.

The purpose of this review is to analyze our current understanding on the host-spectrum, molecular pathobiology of APMV-1 in waterfowls, and host immune responses that may play crucial roles in the disease prevention and control.

## Susceptibility of waterfowls for APMV-1 infection

The Class I APMV-1 isolate was reported in 2006, however, currently these isolates are frequently being reported. Recent epidemiological studies direct that class I APMV-1 are common in domestic waterfowl. All viruses belong to class I are avirulent except JS10-A10 and 9a5b strains [19, 20] which were generated by experimental consecutive passages through chicken. Amongst all genotypes of Class II, genotype I and II are the most prevalent genotypes in the waterfowl and have been isolated from many countries (summarized in Table 1). Waterfowl-origin isolates belonging to genotype VII of APMV-1 are constantly increasing especially in China, Republic of Korea, and Taiwan [16, 21–32]. These emerging outbreaks are increasing the global burden of APMV-1 and causes heavy economic losses [24]. Genotype IV and V have not been isolated from the waterfowl (Table 1). Collectively, these epidemiological studies clearly demonstrate the susceptibility of waterfowls for APMV-1 and their possible roles in the epizootiology of viruses.

**Table 1:** **Field surveillance of ducks and geese for** ***Avian avulavirus 1***

Class	Genotype	Specie/host	Region	Year(s) of isolation	References
I	1	Duck, Mallards Teal	(Zhejiang, Jiangsu, Guangxi, Guizhou, Fujian, Guangdong, Hubei, Guangxi, Shandong, Shanghai) China Sweden (Hyogo) Japan USA (Central and Southern) Finland	2002, 2007, 2008, 2011, 2013, 2015, 2016	[11, 22, 69, 75–79]
I	2	Duck Teal, Mallards	(Florida, Kentucky, Maryland, North Carolina, Pennsylvania, South Carolina, Vermont and Virginia) USA Central and Southern, Finland (Jiangsu, Anhui, Zhejiang, Fujian, Shanghai) China (Chonbuk) Republic of Korea	2009 2006, 2010 2002, 2006, 2007 2007	[54, 77, 80]
I	3	Duck	(Shandong, Jiangsu, Zhejiang, Jiangxi, Guangdong, Shanghai,) China	2004, 2005, 2008, 2009, 2010, 2012, 2013	[21, 63, 68, 81, 82]
I	4	Duck	(Jiangsu) China	2010	[83]
I	5	Duck	(Florida, Kentucky, Maryland, North Carolina, Pennsylvania, South Carolina, Vermont and Virginia) USA	2009	[80]
II	I	Duck Teal, Mallard	(Shandong, Henan, Jiangsu, Anhui, Zhejiang, Anhui, Gaoming, Guangzhou, Guangdong) China (Gyeongbuk, Chonnam, Chungbuk, Chonbuk, Cheju) Republic of Korea (Republic of Sakha) Russia (Island of Öland i.e. Southern part) Sweden Luxembourg Yobe State, Nigeria (Central and Southern) Finland (Tohoku) Japan (Northern Queensland) Australia (South Dakota, Minnesota) USA Taiwan	1995, 2004, 2005, 2006, 2007, 2008, 2009, 2010, 2011	[21, 22, 32, 54, 77, 82, 84–96]
		Pintail	Tohoku, Japan	2003	[97]
II	II	Duck	(Anhui, Yunfu) China Luxembourg Republic of Korea (Tohoku) Japan (North Dakota, South Dakota, Minnesota) USA (Baluchistan, Sindh, Punjab) Pakistan	2005, 2005–2013, 2006–2008, 2006–2009, 2008, 2014–2016	[22, 30, 87, 91, 93, 95, 98]
II	VI	Duck/mallards	Finland	2006	[96]
II	VII	Duck Mallards Black swan, Goose	(Guizhou, Jiangsu, Beijing, Anhui, Guangdong, Yunfu) China (Adygea) Russia Serbia (Gyeongnam) Republic of Korea Taiwan (Punjab, Khyber Pakhtun Khwa) Pakistan	2000, 2005, 2006, 2007, 2008, 2009, 2010, 2014, 2015, 2016	[16, 21–32, 98–101]
II	IX	Duck	(Jiangsu, Beijing, Shandong, Southern China, Guangxi) China	2009, 2010, 2011	[16, 24, 28, 31, 102]
II	XVIII	Duck	Yobe State, Nigeria	2008	[88]
I	1	Geese	(Alaska) Japan USA	1991, 2007	[11, 103]
I	3	Geese	(Jiangsu, Shanghai, Shandong) China	2008, 2009, 2011, 2012	[63]
II	I	Geese	Yobe State, Nigeria	2008	[88]
II	II	Geese	(Jiangsu) China	2003, 2006	[104, 105]
II	III	Geese	(Jiangsu, Guangxi) China	2005, 2006	[106]
II	VI	Geese	(Jiangsu, Guangdong) China	1998, 2013	[18, 107]
II	VII	Geese	(Jiangsu, Guangxi, Anhui, Jilin, Shanghai) China	1997–2001, 2003–2008	[18, 27, 30, 31, 104–106, 108, 109]
II	IX	Geese	China	1997	[31]
II	XII	Geese	China	2010, 2011	[110]

Most of our understanding on the surveillance of APMV-1 in wild birds came from epidemiological studies on avian influenza viruses. Thus, it is required to design APMV-1 dedicated studies to effectively assess the true prevalence of the virus in wild birds. It is essential to understand and establish the foundations to devise control strategies, especially in wild-birds populated and highly vulnerable commercial poultry areas.

## Pathogenicity and special immune responses of waterfowl

**Figure 1:**
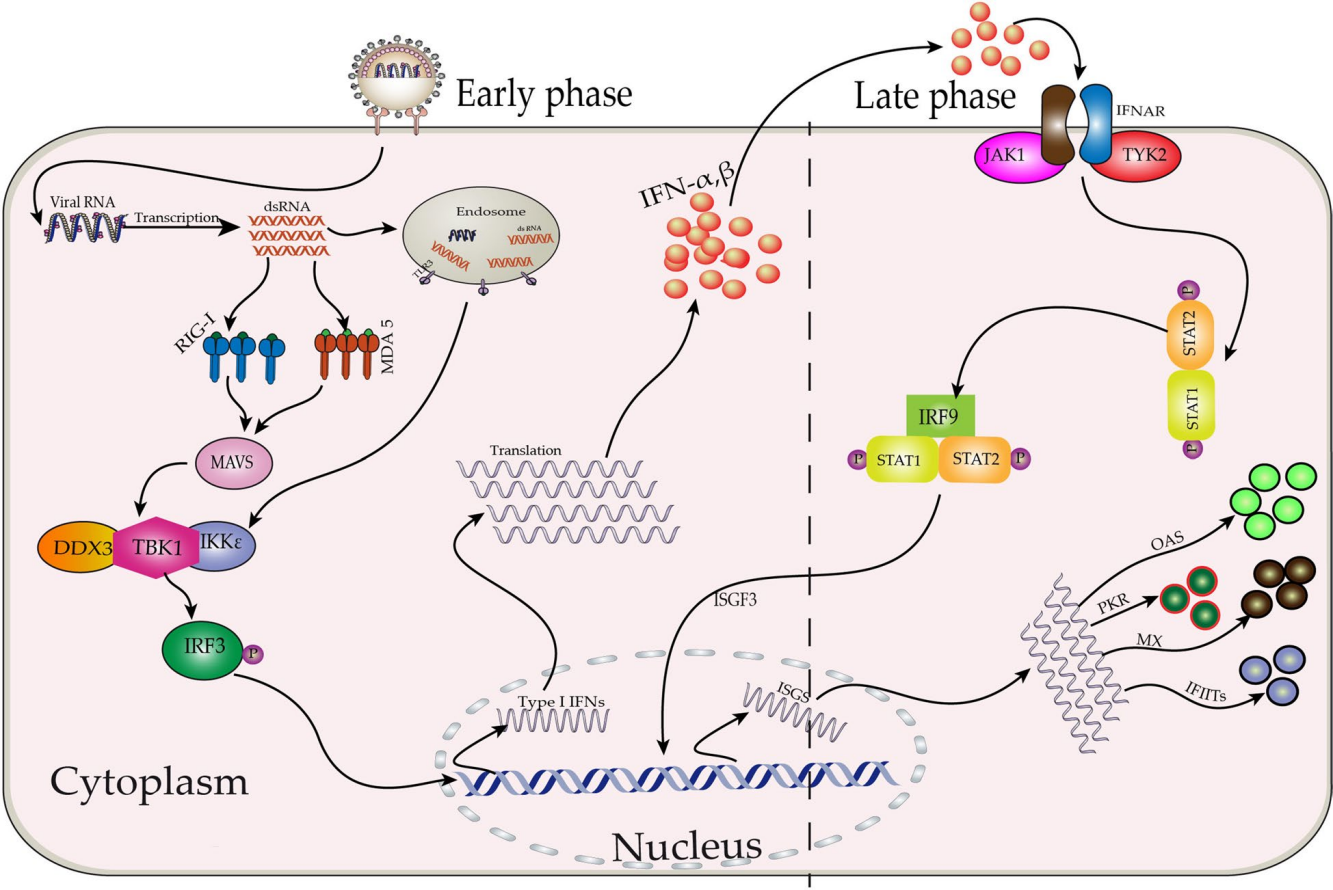
**Pictorial representation of un-inhibited** ***Avian avulavirus 1*** **(APMV-1)-induced type I interferon (IFN) response in waterfowl cells.** After the fusion of virion and plasma membrane, the viral RNA enters the cytoplasm, where it is recognized by retinoic acid-inducible gene I (RIG-I), melanoma differentiation-associated gene 5 (MDA5), or toll like receptor (TLR) 3 and initiates downstream signaling mediated through mitochondrial antiviral-signaling protein (MAVS). Activated MAVS, stimulate the translocation of interferon regulatory factor 3 (IRF3) to the nucleus, leading to the transcription of type I IFNs (IFN-α and β). These upregulations of IFNs may last for is 8–12 h (early phase). Then, these IFNs stimulate JAK–STAT pathway leading to phosphorylation of STAT1 and STAT2 molecules, which (together with factors that are currently unknown in waterfowl) results in the formation of the IFN-stimulated gene factor 3 (ISGF3) transcription factor complex. This multifunctional transcription factor initiates the transcription of hundreds of IFN-stimulated genes (ISGs), which subsequently establish the antiviral state against the invading viruses. Several well-characterized ISGs are revealed in the figure.

Waterfowls are less susceptible to APMV-1 compared to chickens, such as ducks and geese. A key reason for less susceptibility of waterfowl to APMV-1 is the presence of retinoic acid-inducible gene I (RIG-I). RIG-I is absent in the chicken [33] whereas it is present in ducks and goose (Figure 1) [7, 34]. RIG-I and melanoma differentiation-associated gene 5 (MDA5) are the foremost part of retinoic acid inducible gene-like receptors (RLRs) (Figure 1), which senses the cytoplasmic RNA [35]. These sensors can detect the nucleic acids of negative sense RNA such as APMV-1 and influenza viruses [36, 37], resulting in the production of the IFN type I and III, cytokines, chemokines and expression of the antiviral genes [38]. There is a vital role of the RLRs in the recognition of the viruses and antiviral immune responses in macrophages, fibroblast, and dendritic cells [39, 40]. The V protein of APMV-1 blocks the “downstream” signaling pathway by interacting with MDA5 resulting in blockage of strong antiviral response of IFN-β in chicken, which provide the benefit to waterfowl via RIG-I pathway [41, 42]. Positive correlation exist between the resistance to APMV-1 infection and expression of the antiviral genes including RIG-I, IRF3, IRF7 and IFN-β [43] which was further confirmed in a study demonstrating increased expression of RIG-I ultimately leading to decreased APMV-1 load in vitro as well as in vivo [34].

APMV-1 infection increases the expression of interleukin (IL) IL-1β, tumor necrosis factor-α-like factor and interferon (IFN)-β in duck embryo fibroblast (DEF) cells. Distinct innate immune responses of the waterfowl against APMV-1 may reason the resistance of waterfowls to these infections [44]. A higher level of innate immune genes expression has been observed in chicken embryo fibroblast (CEF) compared to the DEF cells [44]. An experimental study reveals that APMV-1 stimulate a strong and intense expression of the IFN-β in ducks compared to chickens [42], and also up-regulate the IFN-β, IFN-regulatory factor 7 (IRF-7), and decreases the virus titer in goose lung and air-sac post-infection [34]. These observations dictate that the strong innate immune responses is a plausible reasons for less susceptibility of duck [42] and geese because its strong protective effects have been revealed to decrease the virus titer in goose-transfected cell [34], which may not be compensated by the IFN-α [33]. Recently, Yang et al. [45] have demonstrated that overexpression of 2′–5′-oligoadenylate synthetase-like gene lessens the replication of APMV-1 in goose cells. In conclusion, waterfowls have diverse innate immunity components, which possibly increase their resistance to the APMV-1 [42].

## Clinical findings

Besides the strong innate immune responses, waterfowl are generally considered long-term carrier of APMV-1 and disease outbreaks have been reported since 1997 [12–14], and were confirmed by follow up experimental studies. Clinically and naturally infected ducks and geese with APMV-1 show clinical signs such as elevated body temperature, excessively excreted oral mucus, dried cloaca, watery, greenish-white diarrhea, vain attempts of eating and drinking, listlessness, anorexia, crouch, eyelid edema and emaciation [14, 23, 46, 47]. Ducks may show up to 70% decrease in egg production, 80% morbidity and 67% mortality [15, 48] however the mortality in ducks varies with the different breeds, virus strain and dose of virus [15]. Some birds also show weakness of legs and wing along with unilateral or bilateral incomplete paralysis and the effects of this paralysis increases with progression of the disease [46]. Duck and geese also show the neurological signs such as muscular trembles, muscular dis-coordination, circling, and twisting of head and neck [23, 46, 48]. These clinical signs disappear according to infection status; mildly affected recover sooner and severely affected birds may recover after 15 days of infection [22, 46].

APMV-1 infected ducks and geese show the gross lesions on the immune organs such as bursa, spleen, thymus, mild to severe tracheitis, kidney enlargement, necrosis of pancreas, congestion on the meninx and in the brain and diffuse brain edema, focal hemorrhages in the mucosa of the proventriculus and intestine (especially duodenum and upper part of jejunum) [14, 22, 23, 46]. Bursal atrophy, hemorrhagic thymus and splenomegaly with white necrotic spots were found in the APMV-1 infected geese and duck [23, 46]. These lesions and histopathological changes may be due to higher viral loads, multi-systemic distribution of the virus in these immune organs [23]. As these immune organs are the reservoir of immune cells, and their destruction may lead to low antibody titer and other infections.

## Experimental infection studies of ducks and geese with APMV-1

Experimental infection studies are necessary to determine the virulence and pathogenesis, in different bird types, age, species, intervention strategies, evaluation, comparison of vaccines etc. Different scientists propose diverse reasons for experimental infection studies and the design of the experimental infection studies varies greatly with the above-mentioned factors. Experimental infection studies on the pathogenesis, infection route, most susceptible age, bird line and immune responses are limited in waterfowl and available information is summarized in Additional file 1 and are briefly discussed below.

Kang et al. [44] have studied the immune related gene expression of chicken and duck embryonic fibroblast (CEF, DEF), by infecting them with APMV-1 of moderate virulent strain NH-10 and highly virulent strain SS-10. Upregulated expression of the Toll like receptor (TLR) 3, TLR7, IL-1β, IL-6, IFN-α, IFN-γ, MHC-I and MHC-II were observed both in CEF and DEF, however, these expression levels were higher in CEF (mechanism is described briefly in Figure 1). Peking duck infection at 3 week of age with NH-10 and SS-10 via intranasal route showed the systemic replication of the virus into small intestine, cecal tonsils, brain, lung, bursa of Fabricius, thymus, and spleen. This study also demonstrated the increased expression of the TLR3, TLR7, RIG-I, MDA5, IL-1β, IL-2, IL-6, IL-8, IFN-α, IFN-β, IFN-γ in lungs compared to thymus. Furthermore, the higher expression level of TLR3, TLR7, IL-1β, IL-2, IL-8, IFN-α, IFN-γ and MHC II were induced by NH-10 than SS-10 in the lungs. Whereas, the expression of the IL-6 and IFN-β in lung as well as thymus was higher for SS-10 group [7]. Similarly, a study of the Zhong et al. [49] has demonstrated the upregulation of the viperin (an IFN stimulated gene) in the DEFs and also in the spleen, kidneys, liver, brain, and blood of Changbai ducks infected with G7 APMV-1 through intranasal or intramuscular route. Experimental infection of geese with genotype VII APMV-1 up-regulate the expression of TLR 1–3, 5, 7, and 15, avian β-defensin 5–7, 10, 12, and 16, IL-8, IL-18, IL-1β, and IFN-γ, and MHC class I in different tissues [50].  

Intranasal inoculation of the Japanese commercial ducks and chicken males with artificially made APMV-1 class I virus 9a5b results in the higher IFN-β in the duck compared to chicken. This study also demonstrated that replication, distribution, tissue damage and apoptosis were more in the immune organs of the chicken compared to duck [42]. Intramuscular infection of different ducks (mallard, Gaoyou, Shaoxing, Jinding, Shanma, and Peking ducks) with JSD0812 strain showed that different strains vary in the susceptibility to the disease [46]. Mallard are the most susceptible and Peking ducks are the most resistant species of birds. Infection of the Gaoyou duck at 15, 30, 45, 60, and 110 with different routes indicate that their susceptibility to disease and virus shedding decreases with the age and birds seldom die after infection through the natural route [46]. Experimental co-infection of ducks with APMV-1, and low or high pathogenic avian influenza virus (LPAIV and HPAIV) indicate that it decreases the virus shedding and transmission to the naïve ducks by contact [51]. Duck after immunization with inactivated vaccine of APMV-1, and challenge with the same live virulent Kenyan APMV-1 resulted in the development of more antibody titer than the unchallenged birds [52]. Experimental infection of geese with virulent APMV-1 genotype VIId and goose origin APMV-1 showed the extensive replication of geese in the immune organs which correlated with the clinical signs and lesions [12, 23] and also it transmission to SPF chickens [12]. Geese and chicken were vaccinated, then infected with goose-origin APMV-1/NA-1 and chicken-origin APMV-1/F48E9, and F48E8 viruses (Additional file 1). Geese are more resistant to F48E8 virus after vaccination. Results indicate that NA-1 vaccine provides a better protection in the form of less morbidity, less mortality and less virus shedding after challenge [53]. Although several natural outbreaks and experimental infections of APMV-1 in waterfowl had been reported. However, it remain to be clarified whether it cause the disease in waterfowl [46]. Inconsistent results in the infection of APMV-1 in waterfowl are due to APMV-1 strain, dose and rout of inoculation, breed, and maternal antibody titer (Additional file 1). Ducks are more resistant to infection through natural route than the geese [46].

In conclusion, these studies indicate that different viruses affect the immune organs and innate immune genes in diverse mechanisms. Waterfowls are more susceptible to APMV-1 infection at an early age, and through natural route results in less damage to immune organs. More comprehensive and detailed studies are warranted for the control of ND in waterfowl.

## Role of waterfowls in emergence of APMV-1

Waterfowl are naturally infected with large number of the viruses which are avirulent and do not cause the diseases in domesticated poultry. Waterfowl are commonly considered to be the natural host as well as carrier of APMV-1 [4]. APMV-1 isolates from the waterfowl are generally lentogenic or potentially pathogenic [54], and may be transmitted to the avian species, leading to increase attention for their role in the transmission/spread and emergence of ND [55–57]. There are increasing concerns about the increased virulence from the lentogenic to mesogenic to virulent pathotypes upon cyclic replication in poultry. These lentogenic isolates may converted to pathogenic viruses through serial passage in susceptible birds [19, 20, 58] and one such isolate have already been documented to be the causative agent of the outbreak in the Ireland [59], but disappeared quickly. All the Class I APMV-1′s had been isolated from the waterfowl, indicating them the natural carrier of these viruses (Table 1). Live bird market (LBM) epidemiological study in United States indicated that from avirulent viruses, 70% belong to class I and 30% belongs to Class II [60]. There is close phylogenetic relationship between the avirulent viruses isolated from the LBM and waterfowl, indicating that APMV-1′s may be transmitted from the waterfowl to the domestic poultry [60, 61]. Transmission of APMV-1 may occur through different routes like, ingestion of the contaminated feed, water air, contaminated feces, animals, humans, contaminated eggs etc. [62]. Mostly domestic waterfowl are reared in semi-closed areas, where they may have the contact with the wild birds and domestic poultry. Therefore, it provides the best natural environment for the spread of APMV-1′s [20, 63].

Waterfowl and shorebirds are not only the host, but are also infected by the APMV-1 and these viruses can also cause the disease in domesticated poultry [55, 56, 64]. Many virulent isolates from the domestic poultry cannot cause disease in waterfowl [21, 53]. Most prevalent virulent genotype VII causing the endemics in China, Japan, Korea [18, 60, 65] are co circulating into the ducks and chicken [66]. This coevolution was further confirmed when the results of Huang et al. [67] declared that some of the circulating APMV-1 had the multiple homologous genomes from chicken, ducks and geese. So, there is dire need to modernize the housing system of waterfowl according to biosecurity point of view to prevent their contact with terrestrial poultry and wild birds. Otherwise, number of virulent [16, 21–29] and avirulent [21, 63, 68, 69] isolates from the waterfowl may easily transmit to commercial poultry farm.

APMV-1 infected waterfowls shed the virus for an extended period of time [24] whereas, the infected chickens clears themselves rapidly and shed virus for short duration [70]. This prolonged virus shedding may facilitate the transmission, persistence and evolved to get some point mutation in the virus [66]. Major issue that should be concerned for virologists is the high evolution rate of some Class I APMV-1 isolates from waterfowls. Pathogenicity of some Class I APMV-1 isolates are constantly increasing and they are naturally converted to low virulent from avirulent viruses (unpublished findings from our lab). This was confirmed by the results of the Meng et al. [20] and Shengqing et al. [19] in which they artificially develop the virulent viruses through serial passage in the air sac and brain of chicken, from avirulent viruses of waterfowl origin. Although the conditions provided in these studies are not naturally existing but recovery of the virus from the air sacs after challenged via nasal or ocular route indicate the possibility of this mechanism.

In conclusion waterfowl plays a vital role in the transmission and re-emergence of ND in terrestrial poultry. It is recommended that rearing facilities of the waterfowl should be separated from commercial poultry.

## Recent advances and challenges for control of Newcastle disease

Cumulating evidences indicate that genetic resistance to APMV-1 exists in various breeds of waterfowl [15]. Comprehensive studies are needed to determine the genetic variability against APMV-1 in different breeds of waterfowls. Genetic resistance of more susceptible breeds can be improved by including the resistant birds in the breeding programs of the commercial waterfowl [6]. APMV-1 resistant breeds should be used to produce highly efficient transgenic poultry birds [6].

Although constant outbreaks of ND have been reported in waterfowl from China and other East Asian countries, however, vaccination against ND in waterfowl is still a matter of debate. There are two consortia of scientists; one favors the vaccination and, others think that domestic waterfowl should not be vaccinated. These veterinarians have their own views, scientist, in the favors of vaccination argue, that it will decrease the chances of outbreaks and lessons the virus shedding. But, scientists, which are against vaccination argue that it will increase the virus burden on the birds, consequently increase the variation rate. The annual rate of change of virulent viruses could be as high as ten times the rate of change of low virulence APMV-1, suggesting that other selective pressures such as vaccination may accelerate the rate of evolution of virulent viruses [71, 72]. They claim that the rearing conditions of waterfowl could not be changed in future because they require water for breeding. Therefore, it is not plausible to prevent their contact with wild birds, resulting in transfer of the wild bird viruses to domestic waterfowls. Secondly, they claim vaccine will develop the antibody titers that will interact with the virus in the future exposures. These antibody responses may force the viruses for evolution. Our viewpoint supports the group of scientists, which claims that birds should be vaccinated. Because, it had already been established that APMV-1 cause the disease in waterfowl [7, 46, 49, 52] and vaccination prevent the chances of the outbreak. Second most important reason is the prolonged virus shedding by non-vaccinated birds, which may transfer to other poultry species and cause heavy economic losses. Theory, that vaccination will increase the virus burden is not very interesting in case of waterfowl because, without vaccination we have to face the same consequences of the high evolution rate. Waterfowls are naturally infected with large and diverse groups of viruses [73]. Development of the vaccines for waterfowl may require strains of waterfowl origin [53] because some lentogenic vaccines phylogenetically are far away than the infectious virus thus may provide partial protection to waterfowl [74]. Vaccine should also be killed because live vaccine virus replicates in the body and leads to shedding of the virus. Virus shedding of live vaccine may interact with the other APMV-1’s present in the environment or birds and results in recombination and future evolution.

## Conclusions

Waterfowls are not only the carrier but also susceptible to APMV-1. Strong innate immune responses may attribute to the less susceptibility of ND to waterfowl. Viral shedding by waterfowl for prolonged duration may increase the transmission, evolution and emergence of new viral strains. A number of APMV-1 isolates from the waterfowl are reported with high mutation rate, which is an alarming matter and may cause the endemics in future. Pathogenicity of ND is affected by different factors such as type, dose, and inoculation route of the virus, and species and age of birds.

Further studies are needed to explore the mechanism, and its intervention to prevent the virus shedding by the waterfowl for a long period. Continuous studies are also required to monitor the APMV-1 in the waterfowl, which may be the future threat to commercial poultry industry. Studies on the humoral immune responses of waterfowl are crucial to develop better intervention strategies. It is recommended that rearing facilities of the waterfowl including ducks and geese should be separated from chicken and turkey flocks to prevent the virus transmission.

## Additional file

**Additional file 1.** **Experimental infection studies of ducks and geese with Avian Avulavirus 1.** Table summarizes the pathobiological findings and immune responses of waterfowl after experimental challenge with Avian Avulavirus 1.

## Abbreviations

APMV-1: *Avian avulavirus 1*
ND: Newcastle disease
ICPI: intracerebral pathogenicity index
SPF: specific pathogen free
NP: nucleoprotein
M: matrix
F: fusion
HN: hemagglutinin–neuraminidase
P: phosphoprotein
L: large RNA-dependent RNA-polymerase
RNA: ribonucleic acid
IFN: interferon
CEF: chicken embryo fibroblast
DEF: duck embryo fibroblast
TLR: toll like receptor
MAVS: mitochondrial antiviral-signaling protein
IRF: interferon regulatory factor
ISGs: interferon stimulated gene
ISGF: interferon stimulated gene factor
LPAIV: low pathogenic avian influenza virus
HPAIV: high pathogenic avian influenza virus
