# Supplementary material for: Pathobiology of Avian avulavirus 1: special focus on waterfowl
Source: Vet Res. 2018 Sep 19;49:94. doi: 10.1186/s13567-018-0587-x (PMC6148804; doi:10.1186/s13567-018-0587-x)
Supplement: Supplementary file 1 — Additional file 1. Experimental infection studies of ducks and geese with Avian Avulavirus 1. Table summarizes the pathobiological findings and immune responses of waterfowl after experimental challenge with Avian Avulavirus 1. [file 13567_2018_587_MOESM1_ESM.docx]

| **Aim of study** | **Animal/age** | **Infection virus** | **Infection route/dose** | **Pathobiology** | **Immune responses** | **References** |
| --- | --- | --- | --- | --- | --- | --- |
| Effect of APMV-1 on the expression of viperin gene in duck | Changbai ducks/ Two-week-old | live/Inactivated G7 (A/Chicken/Guangdong/2008/G7) | Intranasal or injected/10^6^ EID_50_ | Replication peak at 24 hpi in blood, at 48 hpi in liver, and at 72 hpi in brain, lung, and spleen | ▲viperin expression in DEF^1^ and also in the spleen, kidneys, liver, brain, and blood | [[49](#_ENREF_49)] |
| To determine host innate immune response to APMV-1 infection in vitro | DEF* and CEF† | Duck/CH/GD/SS/10 (SS-10) HV and Duck/CH/GD/NH/10 (NH-10) MV, genotypes VII and IX | In vitro study | ▲SS-10 replicated more efficiently than NH-10 | ▲ expression of TLR3, TLR7, IL-1β, IL-6, IFN-α, IFN- β, IFN-γ, MHC-I, MHC-II, TNF-α-like factor in both CEF and DEF but more for CEF | [[44](#_ENREF_44)] |
| To compare the host innate immune response and pathogenesis after APMV-1 infection | Peking Duck infected at the age of 3 week | Duck/CH/GD/SS/10 (SS-10) and Duck/CH/GD/NH/10 (NH-10) | Intranasal/10^6^ EID_50_ | Replicates in small intestine, cecal tonsils, brain, lung, bursa of Fabricius, thymus, and spleen and the titers were higher for SS-10 as compared to the NH-10 | ▲expression TLR3, TLR7, RIG-I, MDA5, IL-1β, IL-2, IL-6, IL-8, IFN-α, IFN-γ in lungs than thymus, it was less in SS-10 as compared to NH-10  ▲expression IFN-β and IL-6 for SS-10 in lungs and thymus | [[7](#_ENREF_7)] |
| To determine the effect of co-infection of low and highly pathogenic avian influenza with APMV-1 on the pathogenicity, virus shedding and transmission | Pekin Ducks/two weeks | APMV-1 /duck/Vietnam (Long Bien)/78/2002;  LPAIV: A/Mallard/OH/421/1987 H7N8  HPAIV: A/duck/VN/NCVD-672/2011 (H5N1). The APMV-1/duck/Vietnam, Long Bien/78/2002 | intraocular and intranasal/  10^7^ EID_50_ of APMV-1,  10^7.5^ EID_50_ of LPAIV,  10^6^ EID_50_ of HPAIV | Co-infection prevents the transmission to naïve ducks  Virus replication interact with each other | No clinical sign was observed in birds infected with APMV-1 and LPAIV  Co infection decrease the virus shedding of APMV-1  ▲mortality by co-infection APMV-1 and HPAIV | [[50](#_ENREF_50)] |
| To determine the susceptibility of different breeds of duck to APMV-1 | Mallard, Gaoyou, Shaoxing, Jinding,  Shanma, and Pekin duck/ two weeks | JSD0812 (VII genotype) | Intramuscular/ 5 × 10^8^ ELD_50_ virus in a volume of 0.2 mL |  | Mallard ducks were the most susceptible, and Pekin ducks the most resistant | [[46](#_ENREF_46)] |
| To demonstrate the effect of age on the susceptibility of APMV-1 in duck | Gaoyou duck/15, 30, 45, 60, and 110 days | JSD0812 (VII genotype) | Intramuscular, intranasal, intraocular/ 5 × 10^8^ ELD_50_ virus in a volume of 0.2 mL | Virus shedding decreases with the age rate of virus isolations was highest from bursa of Fabricius (30%); next from Harderian gland (25%), pancreas (20%), thymus (20%), and laryngotrachea (17.5%); kidney (10%), spleen (5%), heart (5%), brain (2.5%), lung (2.5%), and small intestine (2.5%) | Severity of the disease was high at 15, 30 week age and intramuscular route | [[46](#_ENREF_46)] |
| To compare the histopathological changes after APMV-1 infection in ducks and chicken | Japanese commercial ducks and white leghorn | 9a5b APMV-1 (Class I, velogenic) | Intranasal/10^8.75^ EID_50_ (in a total volume of 0.1 mL) | ▲ apoptosis in duck spleens, bursa and thymic tissues but less than chicken | Earlier and more intense expression of IFN-β in ducks compared to chicken | [[42](#_ENREF_42)] |
| To determine the effect of immunosuppression (IS), antibody titers and virus challenge on the virus replication and antibody titers | Ducks (Kenya indigenous ducks) | Formalin inactivated and live Kenyan virulent Newcastle Disease virus | Intramuscular | ▲ titer in the liver, kidney, ceacal tonsils, and lungs | ▲ antibody titres of IS and NIS challenged ducks than non-challenged  ▼ in antibody in Non-challenged vaccinated ducks non-immunized ducks did not seroconvert | [[51](#_ENREF_51)] |
| To study the pathogenesis of the APMV-1 in geese | Geese/two week | duck/China (Shan Dong)/SDFC/2010(VIId Strain) | Intranasal/10^8.2^ EID_50_/0.2 mL per bird | ▲Viral RNA was at peak in the bursa, spleen and thymus at 2, 4, 6 dpi respectively  ▲ severe lymphoid depletion and necrosis in the spleen, thymus and bursa | ▼ development in antibody titers to APMV-1 | [[23](#_ENREF_23)] |
| To compare the goose and chicken origin genotype VIId APMV-1 pathobiology in chicken and goose | Geese/30days, chicken/30 days | ZJ1, JS-5-05-Go, XJ-2/97, JS-3-05-Ch | Ocular, nasal, oral/10^5.0^EID_50_ | All the viruses were distributed in the tissues  All the test viruses extensively damage the digestive and lymphoid organs in both chickens and geese | Virus origin did not effected the pathogenesis | [[47](#_ENREF_47)] |
| To investigate how APMV-1 spreads between chickens and geese by using goose origin and chicken origin APMV-1’s | Geese, chicken /two week, (vaccination and challenge at) 6 week | First vaccinated and then challenged with APMV-1 /NA-1(goose origin) and F48E9 (chicken origin) | Vaccine; sub cutaneous/ 0.4 mL  Challenge; Ocular, nasal/10^5^ ELD_50_ of APMV-1 /NA-1; and F48E9 | ▼virus shedding in APMV-1 /NA-1 vaccinated group | Geese are more resistance to F48E9 challenge  NA-1 vaccine decrease the morbidity, mortality and increase the cross reactivity of sera  viral strains compete for susceptible hosts | [[52](#_ENREF_52)] |
| To compare the protective efficiency of live and oil emulsion LaSota, ZJ1 HN | Geese, chicken/two week  (vaccination and challenge at) 6 week | Vaccinated with APMV-1/ZJ1 HN and LaSota, and challenged with JS2/06 | Live vaccine; Ocular, nasal/106 EID50  Oil emulsion; intramuscular/0.4 mL  Challenge; Ocular, nasal/10^5^ ELD_50_ | ▼ virus shedding in APMV-1 /ZJ1HN immunized birds | ▲antibody titer Oil-ZHN immunization and both vaccines protected the birds from clinical disease | [[73](#_ENREF_73)] |
| To characterize the host innate immune response to APMV-1 in goose | Goose/40 days | go/CH/LHLJ/1/06 | Intranasally at 10^6^ ELD_50_ |  | ▲ expression of TLR 1–3, 5, 7, and 15, avian β-defensin 5–7, 10, 12, and interleukin (IL)-8, IL-18, IL-1β | [[110](#_ENREF_110)] |

* DEF= Duck embryo fibroblast

† CEF= Chicken Embryo fibroblast
